# Systemic autoinflammatory diseases

**DOI:** 10.1016/j.jaut.2020.102421

**Published:** 2020-02-01

**Authors:** Julie Krainer, Sandra Siebenhandl, Andreas Weinhäusel

**Affiliations:** AIT Austrian Institute of Technology GmbH, Center for Health and Bioresources, Molecular Diagnostics, Giefinggasse 4, 1210, Vienna, Austria

**Keywords:** Systemic autoinflammatory disease, Periodic fever, Inflammasomes, Innate immunity, Inflammation

## Abstract

Systemic autoinflammatory diseases (SAIDs) are a growing group of disorders caused by a dysregulation of the innate immune system leading to episodes of systemic inflammation. In 1997, MEFV was the first gene identified as disease causing for Familial Mediterranean Fever, the most common hereditary SAID. In most cases, auto-inflammatory diseases have a strong genetic background with mutations in single genes. Since 1997 more than 30 new genes associated with autoinflammatory diseases have been identified, affecting different parts of the innate immune system. Nevertheless, for at least 40–60% of patients with phenotypes typical for SAIDs, a distinct diagnosis cannot be met, leading to undefined SAIDs (uSAIDs). However, SAIDs can also be of polygenic or multifactorial origin, with environmental influence modulating the phenotype. The implementation of a disease continuum model combining the adaptive and the innate immune system with autoinflammatory and autoimmune diseases shows the complexity of SAIDs and the importance of new methods to elucidate molecular changes and causative factors in SAIDs. Diagnosis is often based on clinical presentation and genetic testing. The timeline from onset to diagnosis takes up to 7.3 years, highlighting the indisputable need to identify new treatment and diagnostic targets. Recently, other factors are under investigation as additional contributors to the pathogenesis of SAIDs.

This review gives an overview of pathogenesis and etiology of SAIDs, and summarizes recent diagnosis and treatment options.

## 1 Introduction

Systemic autoinflammatory diseases (SAIDs) are a group of disorders caused by a dysregulation of the innate immune system. As their name suggests, a shared key feature is their systemic pathobiology, which means that symptoms can affect the entire body. They can be viewed as the counterpart to autoimmune diseases, having in common that both are caused by abnormal immune responses. The main distinction is that autoimmune diseases are typically defined by a malfunction of the adaptive immune system while in SAIDs the innate immune system is affected (Table 1). The main cell types of the innate immune system are monocytes, macrophages, and neutrophils whereas the adaptive immune response is mediated by B and T cells. The initial defining criteria for SAIDs are the lack of high-titer autoantibodies or antigen-specific T cells [1] and a seemingly unprovoked systemic inflammation. Additional symptoms include fluctuating degrees of fever, as well as abdominal, articular, and cutaneous signs that may lack specificity, making a clinical diagnosis difficult [2]. The name “autoinflammatory disease” was proposed in 1999 by McDermott et al. [1]. Since its initial definition more than 30 new genes associated with autoinflammatory diseases have been identified, affecting different parts of the innate immune system [3]. This growing number is partly the result of genome wide association studies (GWAS).

In most cases, autoinflammatory diseases have a strong genetic background with mutations in single genes. However, they can also be of polygenic or multifactorial origin, with environmental influence modulating the phenotype [3]. While there are clear differences between autoinflammatory and autoimmune diseases, they also share many similarities. In both groups, the underlying pathological processes are directed against the own body. They are systemic, involve the musculoskeletal system, and both include monogenic and polygenic diseases. The innate immune system plays a role in activating the adaptive immune system by antigen presenting cells. Thus, the innate immune system can trigger B and T cell response, and overt or longterm activation of innate immunity can result in autoimmune diseases [11–13]. Another crucial link between adaptive and innate immunity is IL-1β. It is one of the main effector molecules driving autoinflammatory processes and also acts on the effector cells of the adaptive immune system, B and T cells [14]. The similarities and connections between them led to the discussion if autoinflammatory and autoimmune diseases should be considered as one single group of diseases made up of a large spectrum of immunologic abnormalities with autoinflammatory syndromes at one end and autoimmune diseases at the other end. In this continuum model, immunological diseases are grouped along a spectrum with varying degrees of involvement of adaptive and innate immunity components. They are classified along the spectrum as autoinflammatory disease, polygenic autoinflammatory conditions, mixed pattern diseases or polygenic autoimmune conditions, and autoimmune diseases [15].

## 2 Inflammatory response and inflammasome activation

On a molecular basis, SAIDs result from an aberrant activation of the innate immune system. The innate immune system acts as a first line of defence, providing a large array of evolutionary conserved signalling receptors also called pattern-recognition receptors (PRR) to detect and act against pathogens. These membrane-bound and intracellular PRRs are vital for danger sensing and recognition of highly conserved structures expressed by pathogens (microbial pathogen-associated molecular patterns or PAMPs) and damaged cells (non- microbial damage-associated molecular patterns or DAMPs) [16–18].

Membrane-bound PRRs include *Toll-like receptors* (TLRs) and *C-type lectin receptors* (CLRs) which are characterised by the presence of a carbohydrate-binding domain. Intracellular sensors, including *Nucleotide-binding oligomerization domain* (NOD)-like *receptors* (NLRs), *Absence in melanoma 2* (AIM2)-like *receptors* (ALRs), and Pyrin are part of multimeric protein scaffolds called inflammasomes. Generally, inflammasomes consist of sensor molecules, adapter molecules *apoptosis related speck-like protein containing caspase activation and recruitment domains* (ASC), and caspase-1 as an effector molecule. The components of NLRs are a NOD domain and a C-terminal *leucine-rich repeat* (LRR) domain. The N-terminal domain varies between subgroups. NLRP1 has an additional C-terminal FIIND motif and a *C-terminal caspase recruitment domain* (CARD) domain. The AIM2 inflammasome consists of a Pyrin domain (PYD) and a HIN-200 domain mediating the binding of double-stranded DNA. Pyrin has a N-terminal PYD, a B-box domain, *coiled-coiled domain* (CC), and a C-terminal B30.2 domain [19]. ASC consists of PYD and CARD. It recruits caspase-1 through its CARD domain and activates it through proteolytic cleavage [20]. An exception to this mechanism is the Pyrin inflammasome that indirectly senses pathogen virulence factors modifying RhoA [21]. Even though inflammasomes differ in sensor molecules and activation signals (see Table 2), all mediate activation of caspase-1 [16].

**Fig. 1:**
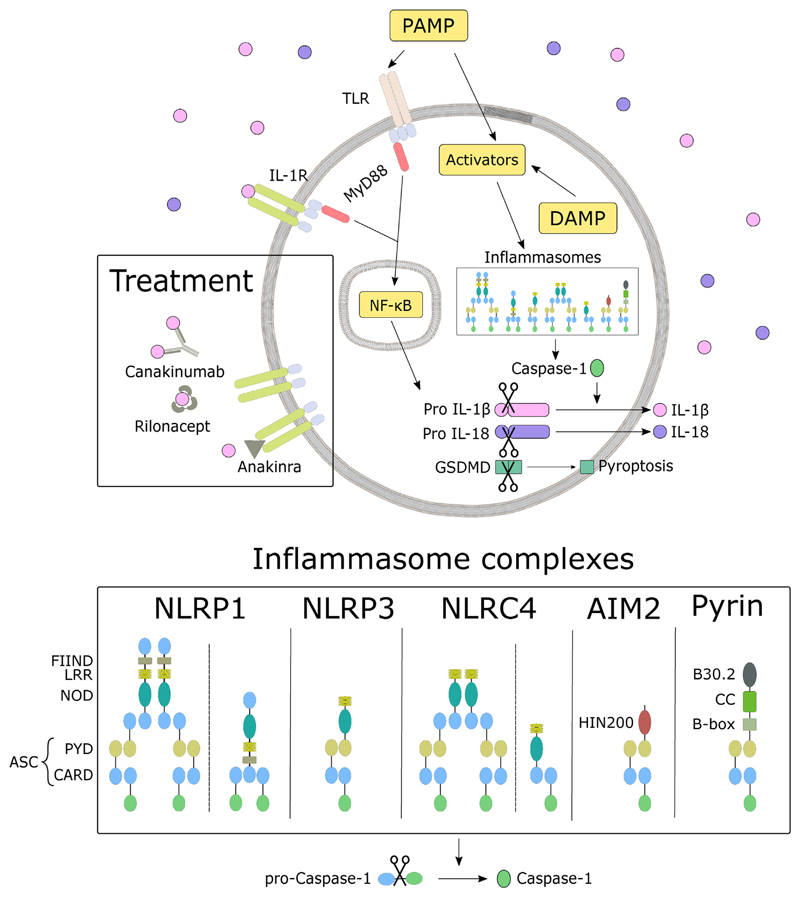
Inflammatory cascade. Upon recognition of PAMP (see Table 2) and DAMP by intracellular sensors, multimeric protein scaffolds called inflammasomes are formed. As a result of this formation, pro-Caspase-1 is activated by proteolytic cleavage and further converting pro IL-1β and pro IL-18 to their bioactive form, which is released into the bloodstream. IL-1 receptors sense IL-1β and reacts by activating the NF-κB pathway. Active NF-κB is translocated to the nucleus where it promotes the transcription of NF-κB-dependent genes, such as NLRP3, pro-IL-β, pro-IL-18, and IL-6. The compositions of the inflammasome complexes are adapted from Ref. [21]. In the treatment section, different IL-1 blocking agents (Canakinumab, Rilonacept) and anti-IL-1 receptor antagonist (Anakinra) are depicted.

Active caspase-1 can catalyze a variety of cellular events crucial for regulating the immune response and inflammation such as the secretion of essential cytokines. The ability of caspase-1 to convert pro-IL-1β into its bioactive form IL-1β was identified in 1989 [25]. Later it was shown that caspase-1 also cleaves IL-18 [26]. Both bioactive forms are released from the cytoplasm into the bloodstream. Once released, IL-1β binds to the IL-1 receptor type I (IL-1RI) which is ubiquitously expressed. Upon binding, the myeloid differentiation primary response gene 88 (MyD88) induces the translocation of active NF- κB to the nucleus where it promotes the transcription of NF-κB-dependent genes, such as NLRP3, pro-IL-β, pro-IL-18, and IL-6, leading to induction and maintaining of the inflammatory response [22,27–29]. The inflammatory cascade is shown in Fig. 1.

Furthermore, caspase-1 cleaves the *membrane-pore-forming protein gasdermin-D* (GSDMD), inducing pyroptosis. Pyroptosis is a rapid inflammatory form of cell death leading to a swelling of the cell and eventually membrane rupture to release alarmins in the extracellular space [19]. Contrary to pyroptosis, apoptosis is a regulated and controlled process that avoids eliciting inflammation. Mediated by a subset of caspases it leads to the formation of membrane-enclosed cellular fragments or apoptotic bodies [30].

In SAIDs many aspects of the inflammatory pathway can be dysre-gulated. A more detailed look on disease specific pathogenic mechanisms are discussed below.

## 3 Pathogenesis and etiology

Genetically, SAIDs can be categorized as monogenic or polygenic/ multifactorial. Monogenic SAIDs are caused by highly penetrant genetic variants in single genes and follow a clear pattern of Mendelian inheritance [31]. Polygenic or multifactorial SAIDs, like systemic juvenile idiopathic arthritis (SJIA), are more complex. They arise from permutations and combinations of common gene variants, where each variant alone confers only a small risk but together or with other extraneous influences becomes pathogenic [32,33].

Overall, polygenic and multifactorial SAIDs are more common than monogenic SAIDs, excluding the eastern Mediterranean basin where the prevalence of Familial Mediterranean Fever (FMF) is highly increased [34]. The most common monogenic SAIDs are FMF, NLRP3-associated autoinflammatory disease (NLRP3-AID; formerly known as Cryopyrin associated periodic syndromes - CAPS), and TNF receptor-associated periodic syndrome (TRAPS) [16].

### 3.1 Monogenic systemic autoinflammatory diseases

Generally, autoinflammatory phenotypes can be classified according to their type of mutation (see Table 3). Depending on their molecular mechanism, SAIDs can be separated into in-flammasomopathies or IL-1β-activation syndromes (FMF, NLRP3-AID, MKD, DIRA, DITRA), protein-folding disorders (TRAPS), NF-κB-activation disorders (Blau syndrome), interferonopathies (Aicardi-Goutières syndromes), and other cytokine-signalling disorders and complementopathies (i.e.: paroxysmal nocturnal hemoglobinuria, atypical hemolytic uremic syndrome) [21,35,36]. An overview of the most common monogenic SAIDs can be found in Table 4.

Inflammasomopathies or IL-1 β-activation syndromes are characterised by an elevation of IL-1β due to inflammasome activation. Associated diseases are FMF, NLRP3-AID and Mevalonate kinase deficiency (MKD).

FMF is the most common hereditary autoinflammatory disease (MIM #249100). It is an autosomal recessive disease and mainly affects people living in the eastern Mediterranean basin, hence the name. In 1997, the International FMF consortium identified mutations in the MEFV gene on chromosome 16p as a cause for the disease [37]. MEFV encodes for Pyrin, an important component of inflammasomes that interacts with caspase-1 and other inflammasome components to regulate IL-1β production [24,38]. Even though the identification of MEFV as disease causing for FMF was in 1997, the role of Pyrin has been a subject of debate. Early studies with mice showed that Pyrin acts as an inhibitor of caspase-1 and the authors suggested an anti-inflammatory role for Pyrin [39]. Other studies demonstrated that Pyrin can assemble an inflammasome complex and act pro-inflammatory [40,41]. Later it could be shown that homozygous gain-of-function Pyrin mutations in mice result in Pyrin inflammasome activation and severe inflammatory phenotypes by generating both pyrin-deficient and knock-in mice with mutated human B30.2 domains [42]. In 2016, the mechanism of Pyrin inflammasome activation has been identified. It could be shown that the Pyrin inflammasome is regulated by RhoA-dependent phosphorylation. Phosphorylated Pyrin interacts with chaperone proteins 14-3-3, keeping Pyrin at an inactive state. Dysregulated interaction between 14-3-3 and Pyrin leads to an activated Pyrin inflammasome [43,44]. FMF is characterised by recurrent fever attacks, abdominal pain, chest pain, and arthritis [45]. The diagnosis of FMF often relies on the phenotypical Tel Hashomer [46] or Yalcinkaya-Ozen criteria [47] and can be supported by genetic analysis [48]. In 20% of patients showing a FMF phenotype, a second mutation of the MEFV gene cannot be found [49].

NLRP3-AID is a group of autosomal dominant diseases that is defined by a mutation in the NLRP3 (*nucleotide-binding domain, leucine-rich repeat family, pyrin domain containing* 3) gene located on chromosome 1q44 [50]. The disease group is also known as Cryopyrin associated periodic syndromes (CAPS) but it has been proposed to rename the group to NLRP3-AID, to reflect the name of the gene over its encoded protein [51]. The NLRP3-AID spectrum includes three formerly distinct diseases: familial cold autoinflammatory syndrome (FCAS; MIM #120100), Muckle-Wells syndrome (MWS; MIM #191900), and Neonatal-onset multisystem inflammatory disease (NOMID) or chronic infantile neurologic cutaneous and articular (CINCA) syndrome (MIM #607115). The three diseases differ in severity where FCAS is the milder form, NOMID/CINCA is on the severe side of the spectrum, and the MWS phenotype is moderate. Gain-of-function or single germline mutations in NLRP3 lead to a low binding affinity of cellular cyclic AMP (cAMP) [52], and a disability of CARD8 binding [53] to mutated NLRP3 protein, thus an increased IL-1β secretion. The NLRP3 inflammasome formation enables activation of caspase-1 that can cleave pro-IL-1β and pro-IL-18 to their biologically active forms. Thus, a dysregulation of the NLRP3 inflammasome leads to inflammation. The clinical manifestation of NLRP3-AID is characterised by chronic systemic and organ inflammation, and the systemic features include fatigue, headache, and influenza-like muscle aches. Organ inflammation effects the skin, muscoskeleton, eyes, ears and the central nervous system [54,55].

MKD is a rare autosomal recessive autoinflammatory disease caused by a loss-of-function mutation in the *mevalonate kinase gene* (MVK) on chromosome 12q24.11. The mevalonate pathway produces isoprenoids, which lead to prenylation of RhoA and further phosphorylate Pyrin to interact with 14-3-3 proteins. A dysregulated mevalonate pathway inhibits the RhoA prenylation and thus activating the Pyrin inflamma-some and increased secretion of IL-1β [44,56,57]. MKD has two associated diseases: Hyper-IgD (Immunoglobulin D) Syndrome (HIDS; MIM #260920) and Mevalonic Aciduria (MEVA; MIM #610377). The common symptoms are fever, gastrointestinal symptoms like abdominal pain and vomiting-diarrhoea, skin rashes, lymphadenopathy, hepatos-plenomegaly, arthralgia, myalgia and mucosal ulcers. As MEVA also has additional symptoms like dysmorphic features, growth retardation (pre- and postnatal), ocular, and neurological involvement, it is a more severe form of MKD than HIDS [58,59]. Contrary to the initial publication [60] and what the name implies, measurements of serum Immunoglobulin D (IgD) levels are not a reliable method to diagnose MKD [61–63].

The protein-folding disorder Tumour Necrosis Factor (TNF) Receptor - Associated periodic syndrome (TRAPS; MIM #142680) is an autosomal dominant autoinflammatory disease that was first described as Hibernian fever [64]. Missense mutations in the gene tumor necrosis factor receptor superfamily member 1A (TNFRS1A) located on chromosome 12 are associated with TRAPS affecting receptor folding and trafficking [65]. Most disease-causing mutations are located within exon 2 to 4. The missense substitutions result in a dysregulation of structurally important cysteine-cysteine disulfide bonds leading to misfolded receptors that accumulate in the cell cytoplasm. This further enhances NF-κB activation, reactive oxygen species (ROS) production, and impaired autophagy [66]. Clinical manifestations of the disease are not completely specific and can be strikingly different [67]. A survey of 158 TRAPS patients of the Eurofever/EUROTRAPS international registry (https://www.printo.it/eurofever/) reported that the most common symptoms are fever, limb pain, abdominal pain, and rash seen in more than 63% of the cohort, followed by periorbital oedema (20%) [68]. For TRAPS diagnosis relies on “suspicion” and genetic testing.

### 3.2 Multifactorial systemic autoinflammatory diseases

For multifactorial SAIDs, like SJIA, Behçet disease, and Periodic fever adenopathy pharyngitis (PFAPA), the pathogenesis is still unknown and requires extensive research to elucidate disease causing mechanisms. SJIA is a subgroup of juvenile idiopathic arthritis (JIA; MIM #604302) [69]. It is the most common rheumatic disease in children but the underlying etiological pathway still needs to be identified [70]. The diagnosis is based on elevated biological markers such as IL-18, S100A12, and MRP8/14 [71,72]. Clinically SJIA patients present with JIA typical arthritis and additional symptoms of systemic inflammation like periodic fever, pericarditis, peritonitis, lymphadenopathy, and organomegaly. One life-threatening complication in SJIA is macrophage activation syndrome (MAS) with a prevalence of about 10% and even higher subclinical prevalence [73].

## 4 Diagnosis

For defined monogenic autoinflammatory syndromes, genetic testing has become a standard and gene panel sequencing is available covering the majority of known genetic loci associated with those SAIDs [80,81]. Papa et al. designed a NGS diagnostic panel, including 41 genes related to SAIDs and additional genes reported in the INFEVERS database [82]. Touitou and Aksentijevich proposed three prerequisites for genetic testing: i) evidence for systemic inflammation (elevated C-reactive protein (CRP), erythrocyte sedimentation rate (ESR), and serum amyloid A (SAA)), ii) a likely monogenic disease, and iii) a plausible candidate causal gene. Diseases with multifactorial inheritance multiple factors, including environmental factors, have a cumulative effect on the disease. Therefore, genetic testing cannot identify multifactorial diseases [3]. The diagnosis of SAIDs often relies on suspicion, resulting in a diagnostic delay of up to 7.3 years [83].

For at least 40%–60% of patients with phenotypes typical for SAIDs, a distinct diagnosis cannot be met, leading to undefined or undifferentiated SAIDs (uSAIDs) [3,14,84]. In 2019, the Eurofever Project described the characteristics of 187 patients with uSAIDs, concluding a need for new classification criteria for monogenic SAIDs which should combine genetic and clinical variables, and the need for further research to provide insight into genotype-phenotype relation [85].

For some defined monogenic SAIDs, like FMF, a distinct diagnosis based on clinical criteria is available. The Tel Hashomer criteria are the most widely used criteria for the diagnosis of FMF in adult patients. It includes a set of ten criteria grouped into *Major criteria, Minor criteria,* and *Supportive criteria.* A diagnosis of FMF requires ≥ 1 major criterion; or ≥ 2 minor criteria, or 1 minor criterion plus ≥ 5 supportive criteria [46]. The Yalcinkaya-Ozen criteria enables a diagnosis of children with FMF. It includes the five criteria *Fever, Abdominal pain, Chest pain, Arthritis,* and *Family history of FMF* [47]. They do not necessarily require molecular confirmation. Another classification criterion set, based on the Eurofever/PRINTO projects was published by Gattorno et al., in 2019. The set combines genetic and clinical findings to classify NLRP3- AID, FMF, TRAPS, and MKD patients [86].

In many cases of multifactorial SAIDs clinical criteria are not available nor applicable. For ideal treatment and in order to prevent severe complications such as amyloidosis, destructive arthropathy, organ damage an early and accurate diagnosis is crucial. Differential diagnosis of SAIDs can be difficult due to the non-specific or overlapping symptoms and the lack of universally accepted screening protocols [3]. One characteristic feature of SAIDs are the recurrent fever attacks. Fever can also be caused by more prevalent conditions such as infectious diseases, congenital immune defects, or neoplasms. In patients with SAID, the fever is periodic, alternating between fever attacks and fever-free episodes. In contrast to chronic illnesses, SAIDs are not associated with a progressive deterioration of the patients. Therefore, it is essential to follow patients with recurrent fever attacks for at least six months before settling on the final diagnosis of autoinflammatory disease. Each individual syndrome has specific characteristics such as the type of skin rash and the areas affected by it or the duration and pattern of acute inflammatory episodes. Together they offer clues for the diagnosis and allow for differentiation [83].

## 5 Treatment

Treatment of patients with SAIDs is aimed at supressing the systemic inflammation. For some patients the use of corticosteroids during attacks can be an option for reducing inflammatory attacks. However, they often require higher doses as the disease progresses. Withdrawal of corticosteroids lead to frequent attack relapses or continuous symptoms [49]. Although the underlying mutations and causes for SAIDs vary widely, a common effect shared by almost all of them is the dysregulation of IL-1 signalling. Given its prominent role in innate immunity and inflammasome formation, it is a successful target for treatment. Treatments targeting IL-1 are e.g. Anakinra, Canakinumab, and Rilonacept [87]. Anakinra is a recombinant anti-IL-1 receptor antagonist that inhibits both IL-lα and IL-1β by binding to the IL-1 receptor. Canakinumab is a selective anti-IL-lβ monoclonal antibody. Rilonacept prevents the interaction of IL-lα, IL-1β, and IL-IRa with cell-surface receptors by acting as a soluble decoy receptor that binds to IL-1β [88]. Anakinra and Canakinumab are the most commonly used medications in the therapy of SAIDs. Since 1972 Colchicine has been the main therapeutic for FMF, where two-thirds of the patients respond positively, one-third are partial-responders, and 5–10% are non-responders. Colchicine exerts its anti-inflammatory effect by suppressing of pyrin oligomerization and interfering with neutrophil migration and adhesion [24]. For non-responders that do not show improvement while adapting the colchicine dose, other treatment options are added to the colchicine treatment. In 2019, El Hasbani and colleagues summarized trials and case studies using anti-IL-1 drugs (Anakinra, Canakinumab, and Rilo- nacept), anti-TNF drugs (Etanercept), anti-IL-6 drugs (Tocilizumab), and Janus kinase inhibitors (Tofacitinib). It was shown that those additional treatment options could be beneficial for colchicine non-responders [89]. TRAPS and MKD patients respond to Etanerecept, a recombinant human TNFR2-Fc fusion protein [90,91]. Treatment of SJIA is challenging, due to its heterogenous nature. IL-6 pathway inhibitor (Tocilizumab), IL-1 receptor antagonist (Anakinra), and anti-IL-1β monoclonal antibody (Canakinumab) have been reported to reduce disease activity [92,93].

For uSAIDs no specific drug is available. Currently the treatment consists of nonspecific immunosuppression with corticosteroids and other disease-modifying anti-rheumatic drugs [94].

## 6 Other factors driving SAIDs

The definition of immune related diseases recently departs from the strict separation between autoinflammatory and autoimmune diseases. The inflammatory spectrum also includes polygenic and multifactorial diseases, where the causes are not completely resolved. Recently, autophagy, epigenetics, microbiome dysregulations, and autoantibody signatures are under investigation as additional contributors to the pathogenesis of monogenic or multifactorial SAIDs.

The innate immune system acts as a first line of defense by sensing PAMPs and DAMPs. Autophagy is an intracellular degradation system that delivers cellular stressors like misfolded proteins, damaged organelles, or intracellular microorganisms into the lysosome. It is a crucial mechanism of the innate immune system to regulate inflammation. It could be shown that after stimulation by inducers, autophagy depleted macrophages accumulate damaged mitochondria and produce a high amount of ROS. Both ROS and mitochondrial DNA activate the NLRP3 inflammasome, leading to an excessive inflammation [95]. Pyrin, also known as TRIM20 recruits the autophagic machinery. It could be shown that FMF associated mutations in the MEFV gene alter the capacity to direct autophagy of inflammasome components, leading to increased IL-1β production [96].

Epigenetic changes that alter the expression of genes, like DNA methylation, microRNA (miRNA) expression, or Histone modifications can be efficiently analysed with microarray and NGS based approaches. In 20% of FMF patients, only one mutation in the MEFV gene is identified, leading to the suggestion of additional causes for disease development. In a study comparing methylation levels of the CpG island within the second exon of MEFV between 30 FMF patients and 21 healthy controls in peripheral leukocytes, a small but significant higher methylation level (76% versus 74%) in FMF patients was shown [97]. Another study analysed methylation levels of inflammasome related genes (IL1B, NLRC5, PYCARD, AIM2, and CASP1) in monocytes and monocyte-derived macrophages from NLRP3-AID patients compared to those of healthy controls. The methylation levels in untreated patients were lower than in healthy controls after stimulation with IL-1β. Patients treated with IL-1 drugs had levels similar to healthy controls [98].

miRNA expression is another epigenetic regulator analysed in SAIDs. miRNAs are ~21 nucleotides long, single stranded RNA molecules that regulate host gene expression by base-pair binding to messenger RNA (mRNA). It is known that they are involved in most types of inflammatory responses and contribute significantly to the magnitude of the response by impacting the development of inflammatory cell subsets and by establishing the level of immune cell function [99]. A review by Balci-Peynircioglu et al. summarizes miRNAs associated with FMF, TRAPS, NLRP3-AID, Behçet’s disease, and NOMID, with a strong emphasis on FMF specific miRNAs [100]. Studies demonstrated that during fever attacks in three FMF groups differing in MEFV mutation location (exon 10, exon 3, neither exon 3 nor exon 10), the level of 25 circulating miRNAs changed specifically for the respective group [101]. Another study showed a lower miR-204-3p expression in the serum of FMF patients during an attack inhibiting inflammatory cytokine release via the phosphoinositide 3-kinase γ pathway [102]. In FMF patients without an inflammatory attack miR-132, miR-146a, miR-15a, miR-16, miR-181a, miR-21, miR-223, miR-26a, and miR-34a were lower compared to patients during an active attack [103]. Other studies comparing the expression levels of 798 mature miRNAs in peripheral blood mononuclear cells (PBMCs) of FMF patients and healthy controls showed that miR-144-3p, miR-21-5p, miR-4454, and miR-451a were increased and miR-107, let-7d-5p, and miR-148b-3p were decreased [104]. Apart from miRNA expression analysis in FMF patients, it could be shown that the NLRP3 inflammasome activity is negatively controlled by miR-223 and the expression is associated with NLRP3-AID [105]. miR-223 is a posttranscriptional regulator of NLRP3 expression, and miR-223 deficient mice display increased immune infiltration by neutrophils and monocytes, hyperactivated NLRP3 and IL-1 β release. Upon delivery of miR-223 the inflammation, and cytokine balance could be restored [106]. Comparing TRAPS patients to controls also showed altered circulating miRNA levels, where miR-134, miR-17-5p, miR-498, miR-451a, miR-572, miR-92a-3p are downregulated, and miR-150-3p, miR-92a-3p, miR-22-3p, miR-30d-5p are upregulated [107].

The above-mentioned studies and discoveries show the importance of autophagy and epigenetic factors that could act as novel diagnostic and treatment targets.

Another important factor is the human microbiome that is acquired after birth and shaped by environmental factors and the host immune system. The composition of gut microbiome is important for health and disease and dysbiosis has a central role in the pathogenesis in patients with inflammatory bowel disease (IBD). As NLRs sense microbial activation signals (shown in Table 2), they are important in controlling the bacterial community in the intestine [108]. A study comparing the gut microbiome of FMF patients against healthy controls, showed a specific shift in composition of bacteria, where diseased patients had a reduced amount of bacterial diversity [109]. Further studies are required to investigate the influence of the microbiome in SAIDs.

Autoantibodies play a crucial role in early diagnosis and classification in autoimmune diseases [110]. SAIDs are known to lack high-titer autoantibodies [1] but as immune related diseases are placed on a spectrum between autoimmune and autoinflammatory diseases [15], like the multifactorial disease SJIA, the role of autoantibodies needs to be reconsidered. Systematic profiling using human proteome arrays presenting immobilized recombinantly expressed human proteins can be used like in cancer studies (e.g. [111,112]) to elucidate antibodyprofiles at an “immunomics”-level. Additionally, proteomics and metabolomics can be used to identify molecular-pathological layers in SAIDs in the near future by cooperative research.

## 7 Conclusion

Systemic autoinflammatory diseases (SAID) are a growing group of disorders caused by a dysregulated activation of the innate immune system. Most autoinflammatory diseases are related to the activation of the Interleukin-1 pathway. The pathogenesis of these conditions is often driven by mutations in genes encoding proteins that are involved in the assembly of inflammasomes [24]. Since the first discovery of mutations in MEFV as a cause for FMF in 1997, the number of identified diseases, genes, and mutations responsible for those diseases has increased, resulting from advances in technology (e.g. genome wide association studies). Even though FMF is the best described SAID, there are still several unanswered questions. Based on the NIH, 52 clinical trials for “autoinflammatory disease” are currently active (https://clinicaltrials.gov/) [113] in order to find new treatment options for these severe and often life threatening diseases.

Disease-causing mutations can be identified for only half of the patients with SAIDs and the other half is classified as undefined or undifferentiated SAIDs. Improving the clinical definitions and creating precise diagnostic criteria for autoinflammatory disorders is important in order to better understand the differences between diseases and for more efficient therapies [114]. The implementation of a disease continuum model combining the adaptive and the innate immune system with autoinflammatory and autoimmune diseases as described in McGonagle et al. [15] shows the complexity of the diseases and the importance of new methods and international collaboration to elucidate molecular changes and causative factors in SAIDs. The timeline from onset to diagnosis takes up to 7.3 years [83] highlighting the indisputable need to identify new treatment and diagnostic targets. These novel targets could be identified using methods to detect differences in autophagy, DNA methylation, microRNA expression, microbiome composition, autoantibody signatures, and others.

Due to global transition, the number of SAIDs that have a high prevalence in specific countries could increases in other countries, making clinical awareness in diagnosis crucial. This awareness can be achieved with the work of scientific societies or networks such as listed below, and by conducting active research in this area.

European Scientific Societies and Networks:

- ERN RITA: European Reference Network: Immunodeficiency, Autoinflammatory and Autoimmune Diseases (http://rita.ern-net.eu/)
- PRES: Paediatric Rheumatology European Association (https://www.pres.eu/)
- PRINTO: Paediatric Rheumatology International Trials Organisation (https://www.printo.it/)
- ESID: European Society for Immunodeficiencies (https://esid.org/)
- ISSAID: International Society of Systemic Auto-Inflammatory Diseases (https://issaid.umai-montpellier.fr/)

## Supplementary Material

**Appendix A. Supplementary data**

Supplementary data to this article can be found online at https://doi.org/10.1016/j.jaut.2020.102421.

Supplementary Data

## Figures and Tables

**Table 1:** *Differences between autoinflammatory and autoimmune diseases* [4–10].

	Autoinflammation	Autoimmunity
Immune dysregulation	Innate immune system	Adaptive immune system
Predominant cell types	Monocytes, macrophages, neutrophils	T cells, B cells
Cytokine targets used therapeutically	TNF, IFNαβ, IL-1, IL-2, IL-12, IL-23, IL-18	IFNγ, TNFα, IL-1, IL-2, IL-4, IL-6, IL-5, IL-9, IL-10, IL-12, IL-13, IL-17, IL-22, IL-23
Pathogenesis of organ damage	Neutrophil- and macrophage-mediated	Autoantibody- or autoantigen-specific T cell-mediated

**Table 2:** *Inflammasome sensor proteins, their corresponding activating signals, and structure* [22–24].

Inflammasome complexes	Structure	Activating signals
NLRP1b	CARD - NOD - LRR - FIIND - CARD	Key virulence factors of Bacillus anthracis
NLRP3	PYD - NOD - LRR	Diverse endogenous danger signals and pathogenic molecules
NLRC4	CARD - NOD - LRR	Bacterial flagellin, type III secretion system components
AIM2	PYD - HIN200	Direct binding of double-stranded DNA
Pyrin	PYD - B-box - CC - B30.2	Detection of bacterial modifications of Rho GTPases

**Table 3:** Categorization of causative mutations for autoinflammatory disorders [27,28] Abbreviations: MKD: Mevalonat kinase deficiency; DIRA: deficiency of the IL-1 receptor antagonist; DITRA: deficiency of IL-36 receptor antagonist; PLAID: PLCγ2-associated antibody deficiency and immune dysregulation; APLAID: autoin flammation and PLAID; LAID: LYN-associated autoinflammatory disease.

Type of mutation	Effect	Disease
Gain-of-function mutations in genes encoding PRRs or their adaptor molecules	constitutively increased innate immune sensor function; increased or prolonged production of proinflammatory mediators	FMF, NLRP3-AID, Aicardi-Goutières syndromes, Blau Syndrome
Loss-of-function mutations or haploinsufficiency of molecules controlling cell homeostasis	accumulation of intracellular stressors that stimulate intracellular sensor/PRR activation and the production of proinflammatory mediators	TRAPS, MKD
Loss-of-function mutations of negative regulators that downregulate proinflammatory responses	loss-of-function of a cytokine receptor antagonist or anti-inflammatory cytokine; failure to terminate the release of inflammatory mediators by inflammatory cells	DIRA, DITRA
Mutations that alter immune receptor signaling	Hyper-responsiveness to immune signals; increased signaling through receptors controlling innate immune cell function (the resulting diseases often have more complex phenotypes with overlapping features of autoinflammation, immunodeficiencies and autoimmunity)	PLAID/APLAID, LAID, Cherubism

**Table 4:** Overview of the most common hereditary monogenic SAIDs. Abbreviations: AR: autosomal recessive; AD: autosomal dominant.

Disease		OMIM	Affected Gene	location	reported INFEVERS variants	Inheritance	Prevalence	Male/female ratio	Treatment	Mechanism
**FMF**		#249100	MEFV	16pl3.3	365	AR	Turkey 1:4000-1:1000 [74] Israel 1:1000 (in non-Ashkenazi Jews) Armenia 1:500 [38]	1:1 [75]	Colchicine IL-1 inhibition	Inflammasomopathy
**NLRP3-AID**	*FCAS* *MWS* *NOMID*	#120100 #191900 #607115	NLRP3	lq44	227	AD	France 1:360000 [76]	2:1 [77] 1:1 [77] 1:1 [77]	IL-1 inhibition IL-1 blockage NSAIDs/Corticosteroids (primary maintenance therapy)	Inflammasomopathy
**MKD**		#260920	MVK	12q24.11	227	AR	Netherlands 5:1000000 [78]	1:1 [79]	IL-1 blockage IL-6 blockage TNF-α blockage NSAIDs/glucocorticoids (symptom relief during inflammation) Etanercept	Inflammasomopathy
**TRAPS**		#142680	TNFRS1A	12pl3.31	163	AD	1:1000000 [68]	3:2 [66]	IL-1 blockage Etanerecept NSAIDs/Corticosteroids (primary maintenance therapy)	protein folding disorder
